# Undifferentiated Purpuric and Desquamative Drug Eruption After Pembrolizumab and Empagliflozin Exposure: A Clinicopathologic Diagnostic Pitfall

**DOI:** 10.7759/cureus.106689

**Published:** 2026-04-08

**Authors:** Navanita Biswas, Imran Khokhar, Jian J Fu

**Affiliations:** 1 Internal Medicine, Reading Hospital, Tower Health, West Reading, USA; 2 General Surgery, Bronx Lebanon Hospital, New York, USA; 3 Pathology, Reading Hospital, Tower Health, West Reading, USA

**Keywords:** cutaneous vasculitis, immune-checkpoint-inhibitor, leukocytoclastic vasculitis (lcv), pembrolizumab., purpuric eruption

## Abstract

Pembrolizumab can be associated with immune-related cutaneous adverse events, including rare purpuric eruptions that may pose a diagnostic challenge when clinicopathologic findings are inconclusive. While pembrolizumab is generally well tolerated, immune-related adverse events (irAEs) can affect multiple organ systems. Vasculitis is a rare irAE, with heterogeneous clinical presentations ranging from cutaneous purpura to life-threatening systemic involvement.

We report the case of a 75-year-old male with a history of myelodysplastic/myeloproliferative neoplasm (MDS/MPN) overlap syndrome and stage IIIA lung adenocarcinoma who developed a widespread purpuric eruption with desquamation shortly after the first cycle of pembrolizumab and subsequent exposure to empagliflozin. Infectious workup was negative. Skin biopsy revealed full-thickness epidermal necrosis with re-epithelialization and mild superficial perivascular lymphocytic infiltrate, findings not typical for leukocytoclastic vasculitis (LCV), which usually shows neutrophilic infiltration, leukocytoclasia, and fibrinoid necrosis of vessel walls. The differential diagnosis included immune-mediated small-vessel vasculitis, severe drug eruption, and Stevens-Johnson syndrome/erythema multiforme spectrum, with empagliflozin representing a significant competing potential trigger given the close temporal relationship to rash onset. The atypical histopathology may be attributed to delayed biopsy and prior systemic corticosteroid treatment, which can obscure classic features of LCV. Pembrolizumab was discontinued, and the patient improved with corticosteroids.

This case highlights the diagnostic challenge of evaluating a purpuric and desquamative drug eruption in a patient with competing medication exposures, particularly when biopsy findings are nonspecific or obtained late in the disease course. Clinicians should maintain a high index of suspicion for serious cutaneous adverse reactions in patients receiving immune checkpoint inhibitors who develop purpuric rashes, even in the absence of classic histopathologic features. Early dermatologic evaluation, timely biopsy, and appropriate specimen handling for direct immunofluorescence are critical to guide diagnosis and management. Given the nonspecific histopathologic findings, delayed biopsy, concurrent corticosteroid use, and competing medication exposure, definitive attribution to pembrolizumab could not be established.

## Introduction

Pembrolizumab is an immune checkpoint inhibitor (ICI) targeting PD-1 and has shown efficacy in several cancers, including non-small cell lung cancer [1,2]. However, by enhancing the immune response, pembrolizumab can also cause immune-related adverse events (irAEs).

Skin toxicities are among the most common adverse effects seen with ICIs, ranging from mild maculopapular eruptions to severe inflammatory dermatoses. In clinical practice, these cutaneous reactions often demonstrate significant overlap in both morphology and histopathologic findings, making definitive diagnosis challenging.

One reported manifestation is leukocytoclastic vasculitis (LCV), a small-vessel vasculitis that typically presents as palpable purpura involving the lower extremities and is characterized histologically by neutrophilic infiltration, leukocytoclasia, and fibrinoid necrosis [3]. However, similar purpuric or desquamative eruptions may also be seen in severe drug eruptions and Stevens-Johnson syndrome (SJS)/toxic epidermal necrolysis (TEN)-like reactions, particularly in patients receiving multiple medications.

Importantly, the distinction between these entities in real-world settings is frequently limited by imperfect diagnostic conditions. Delayed biopsy, prior corticosteroid exposure, and inadequate specimen handling for direct immunofluorescence (DIF) may significantly alter or obscure classic histopathologic features, resulting in nonspecific or overlapping findings.

Additionally, patients receiving ICIs are often exposed to multiple concomitant medications, introducing potential confounding drug-related etiologies that further complicate attribution of causality.

Although ICI-associated vasculitic and purpuric eruptions have been reported, these entities are uncommon and may not present with classic or distinguishable features, particularly when evaluated outside ideal diagnostic conditions. Increasing evidence suggests that clinicopathologic discordance is not uncommon, and overlapping features between vasculitis, drug eruption, and SJS/TEN-like reactions may occur.

In this context, we present a case of a purpuric and desquamative eruption developing during pembrolizumab therapy in the setting of concurrent medication exposure, in which the distinction between cutaneous vasculitis and severe drug eruption remained diagnostically challenging.

## Case presentation

A 75-year-old man with a complex medical history, including a myelodysplastic/myeloproliferative neoplasm (MDS/MPN) overlap syndrome managed with pacritinib and a recent diagnosis of stage IIIA pulmonary adenocarcinoma, was initiated on pembrolizumab. Due to persistent cytopenias and underlying bone marrow dysfunction, he was not considered an appropriate candidate for standard platinum-based chemotherapy.

Shortly after receiving his first dose of pembrolizumab (200 mg IV), the patient developed low-grade fevers and diffuse myalgias. A chest computed tomography (CT) scan revealed new diffuse ground-glass opacities predominantly involving the left lung. While ICI-related pneumonitis was considered, the pulmonary team attributed the findings to radiation-induced pneumonitis, given the patient’s recent completion of thoracic radiation (30 fractions). The patient was started on prednisone 60 mg daily, followed by a prolonged taper over approximately three months. High-dose corticosteroids led to improvement in symptoms and radiographic findings. Radiation oncology noted that although radiation pneumonitis typically manifests within 2-4 months post-treatment, the addition of pembrolizumab may have accelerated the inflammatory response.

**Figure 1:**
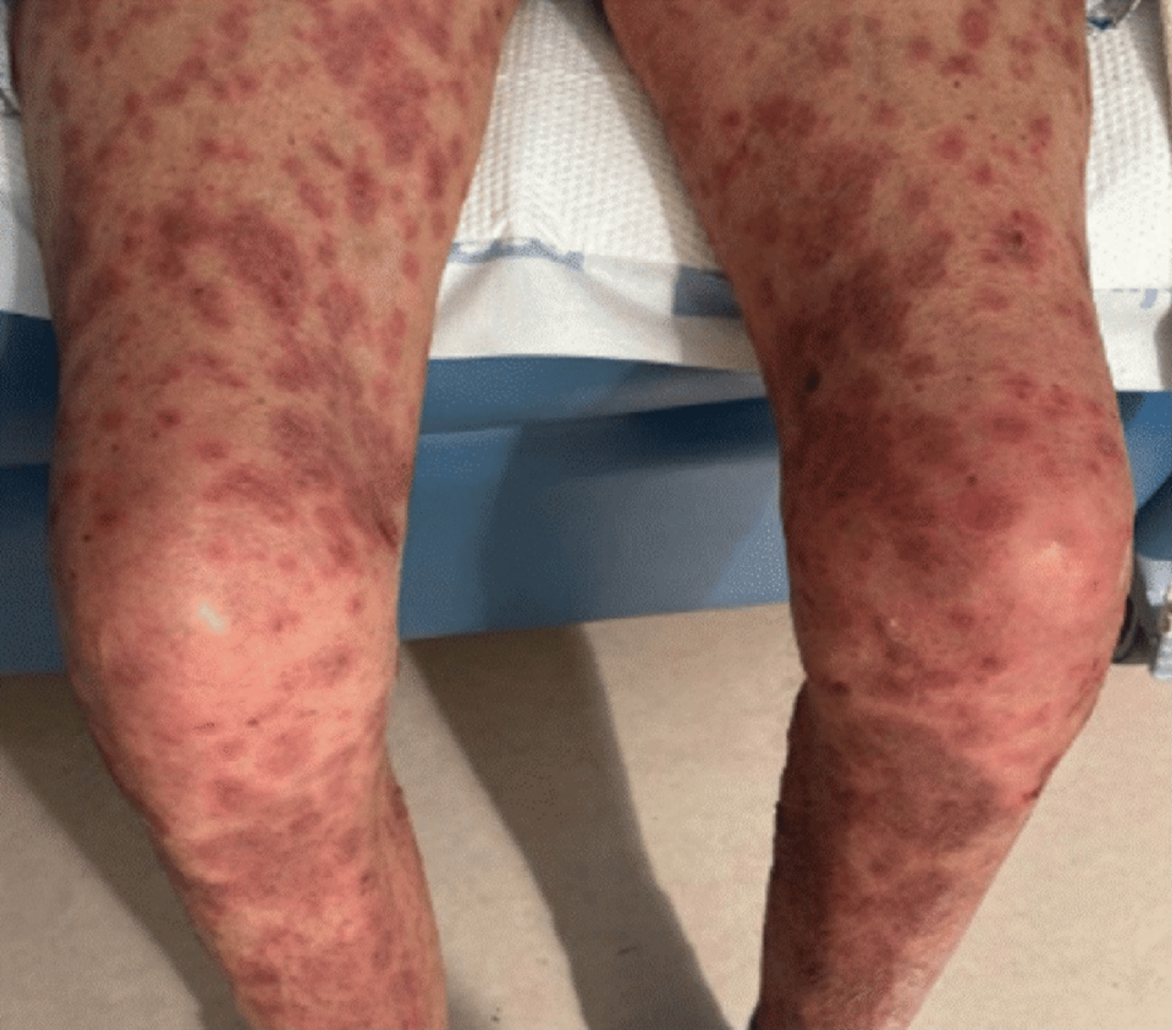
Purpuric Eruption of the Bilateral Lower Extremities Clinical photograph showing a palpable purpuric rash involving both lower extremities.

Approximately three weeks later, the patient was found to have a newly reduced left ventricular ejection fraction (LVEF) of 30%, raising concern for ICI-associated myocarditis or cardiomyopathy. He was started on guideline-directed medical therapy, including a beta-blocker, sacubitril/valsartan (Entresto), and empagliflozin (Jardiance). Empagliflozin was initiated for the first time (no prior exposure). Within 48 hours of starting empagliflozin, the patient developed a generalized pruritic rash with purpuric features and areas of desquamation, without mucosal involvement. The rash was described as palpable, non-blanching, painless, and more prominent over the bilateral lower extremities (Figure 1), although it involved multiple body areas. Empagliflozin was promptly discontinued due to concern for a hypersensitivity reaction. However, the rash progressed despite drug cessation. A skin biopsy was obtained approximately three weeks after rash onset, during which time the patient remained on systemic corticosteroids. At the time of biopsy, the patient was receiving prednisone 20 mg daily. DIF testing was not performed, as the biopsy specimen was placed in formalin, which precludes immunofluorescence analysis.

**Figure 2:**
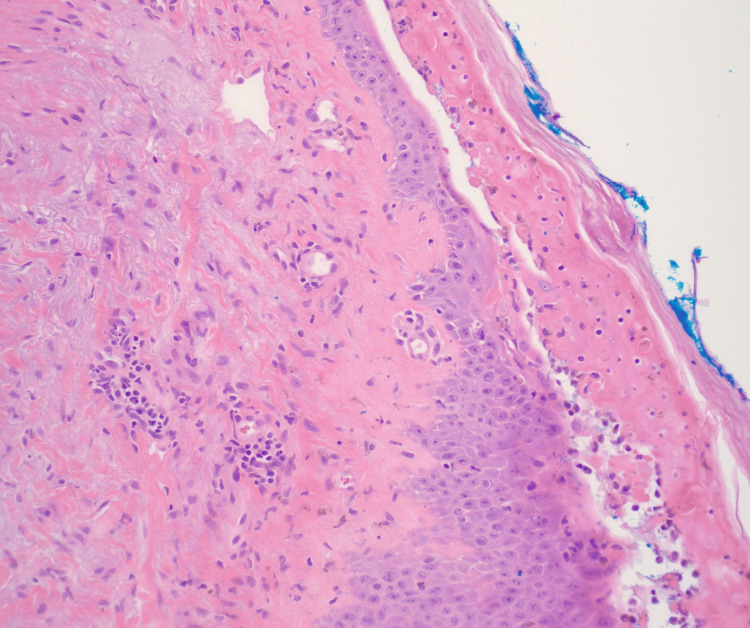
Skin Biopsy Showing Epidermal Necrosis and Superficial Perivascular Inflammation High-power photomicrograph (20×, hematoxylin and eosin stain) demonstrating full-thickness epidermal necrosis with re-epithelialization and mild superficial perivascular lymphocytic infiltrate.

Histopathologic examination revealed full-thickness epidermal necrosis with re-epithelialization and a mild superficial perivascular lymphocytic infiltrate (Figure 2). These findings were nonspecific and not consistent with classic LCV, which typically demonstrates neutrophilic infiltration, leukocytoclasia, and fibrinoid necrosis of vessel walls. Instead, the observed features may be more compatible with a severe drug eruption or an undifferentiated inflammatory cutaneous process. However, the delay in biopsy timing and concurrent corticosteroid therapy may have altered histologic features, potentially masking diagnostic findings.

The differential diagnosis initially included SJS and severe cutaneous drug eruption. Although the absence of mucosal involvement made classic SJS less likely, severe drug eruption and erythema multiforme spectrum remained important considerations. Negative infectious and autoimmune serologies, including ANA, ANCA, HIV, and hepatitis panels, and modestly elevated inflammatory markers (C-reactive protein 1.7 mg/dL, low complement C3 at 50 mg/dL with normal C4) supported a medication-associated inflammatory process. Laboratory findings are summarized in Tables 1-4.

**Table 1:** Complete Blood Count MCV, mean corpuscular volume; MCH, mean corpuscular hemoglobin.

Parameter	Patient Value	Reference Range	Units
White Blood Cells	5.9	4.8-10.8	×10³/μL
Red Blood Cells	2.8	4.50-6.10	×10⁶/μL
Hemoglobin	7.8	14.0-17.5	g/dL
Hematocrit	24.5	39-58	%
MCV	87.5	80-99	fL
MCH	27.9	27-34	pg
MCH Concentration	31.8	31-37	g/dL
Platelets	89	130-400	×10³/μL

**Table 2:** Coagulation Profile INR, international normalized ratio.

Parameter	Patient Value	Reference Range	Units
INR	1.3	0.9-1.1	
Prothrombin Time	16.1	11.7-14.5	Seconds
Partial Thromboplastin Time	29	22.8-34.2	Seconds

**Table 3:** Comprehensive Metabolic Panel BUN, blood urea nitrogen.

Test	Result	Reference Range	Units
Sodium	142	135-145	mmol/L
Potassium	3.7	3.5-5.0	mmol/L
Chloride	107	98-106	mmol/L
CO₂ (Bicarbonate)	27.3	22-29	mmol/L
BUN	35	7-20	mg/dL
Creatinine	1.37	0.6-1.3	mg/dL
Glucose (Fasting)	121	70-99	mg/dL
Calcium	7.9	8.5-10.5	mg/dL
AST (SGOT)	8	10-40	U/L
ALT (SGPT)	8	7-56	U/L
Total Bilirubin	0.4	0.1-1.2	mg/dL

**Table 4:** Additional Laboratory Testing

Test	Result	Reference Range	Units
ANCA Screen	Negative	Negative	
ANA Screen	Negative	Negative	
Complement C3	50	82-185	mg/dL
Complement C4	18	15-53	mg/dL
Proteinase Antibody	<1	<1	AI

**Figure 3:**
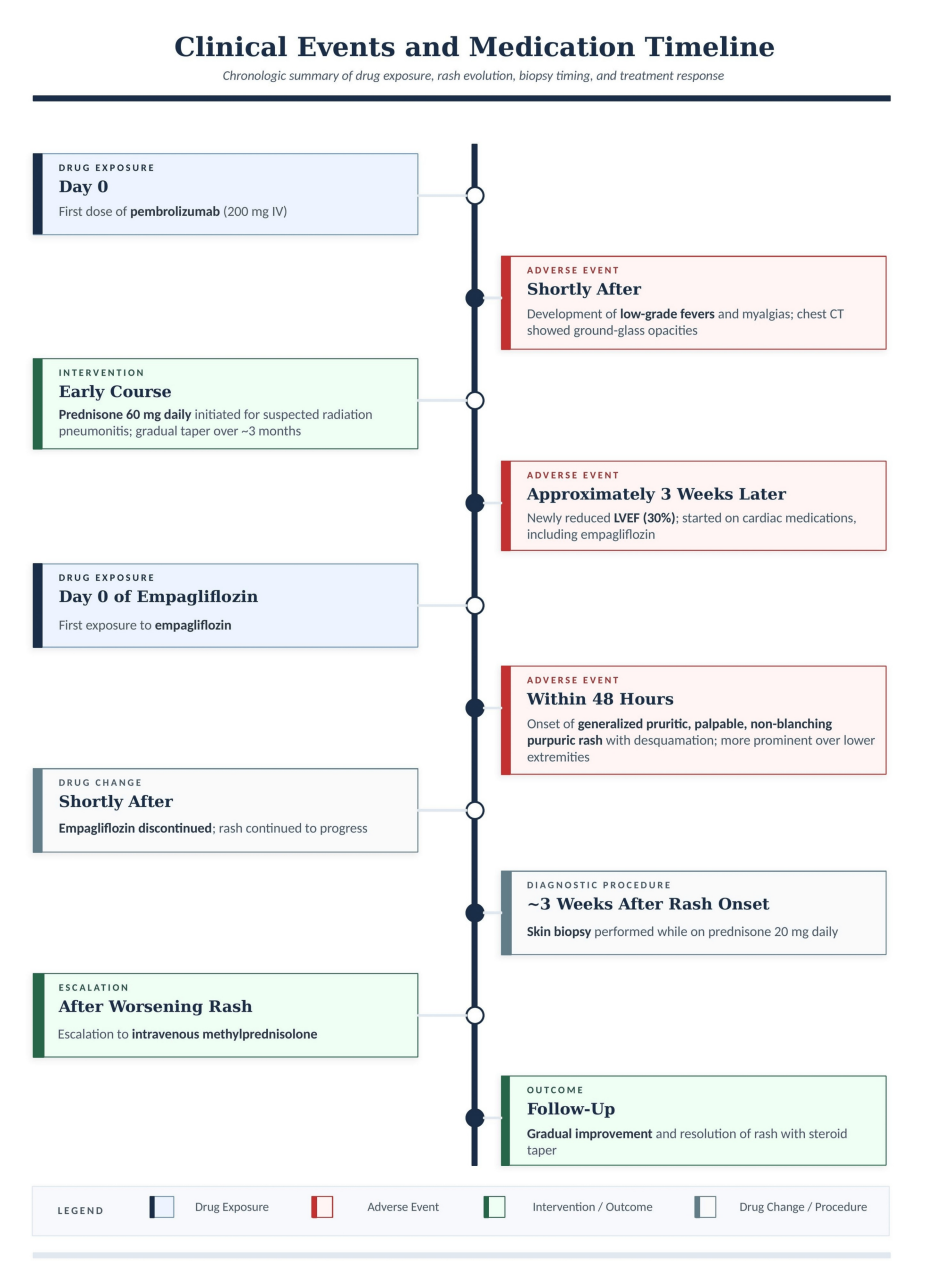
Clinical Events and Medication Timeline Timeline showing pembrolizumab exposure, corticosteroid treatment, empagliflozin initiation, rash onset and progression, biopsy timing, treatment escalation, and clinical improvement.

Given the worsening cutaneous findings and overall clinical context, the patient’s corticosteroid regimen was escalated to intravenous methylprednisolone, followed by a tapering course of oral prednisone. This led to gradual resolution of the rash. The clinical improvement with corticosteroid therapy supported an immune-mediated cutaneous adverse event; however, due to nonspecific histopathology, delayed biopsy, concurrent corticosteroid use, absence of DIF, and competing drug exposures (particularly empagliflozin with a strong temporal relationship), the eruption was best characterized as an undifferentiated purpuric and desquamative drug eruption with competing etiologies rather than confirmed pembrolizumab-induced LCV. The clinical and medication timeline is summarized in Figure 3.

## Discussion

LCV represents a small‑vessel vasculitis marked histologically by neutrophilic infiltration, fibrinoid necrosis, and leukocytoclasia, typically manifesting as palpable purpura, most often on the lower extremities. While most cases are idiopathic or triggered by infection, autoimmune disease, or medications, ICIs are increasingly recognized as rare but significant causative agents.

Pembrolizumab, an anti-PD‑1 monoclonal antibody, is widely used in oncology, but like other ICIs, it can trigger irAEs across multiple organ systems. Although common cutaneous toxicities include rash and pruritus, reports of cutaneous vasculitis are sparse.

An observational, retrospective pharmacovigilance study identified 82 cases of vasculitis among 31,321 adverse events (0.26%) reported in patients receiving ICIs, with a mortality rate of 6.1% [4]. Among reported vasculitis subtypes, small-vessel cutaneous manifestations such as LCV appear to be less frequent than large-vessel or neurologic forms. Another study reported that the incidence rate of LCV was 4.5 per 100,000 person-years (95% CI, 3.5-5.4) [5].

The exact mechanisms underlying ICI-induced LCV remain speculative. Experimental models point toward disruption of the PD-1/PD-L1 axis, leading to unchecked T-cell activation, endothelial injury, and vascular inflammation. In giant-cell arteritis models, PD-1 blockade exacerbated vascular inflammation with cytokine upregulation (e.g., IL-6, IFN-γ, and TNF-α) and intimal hyperplasia. Similar pathways may underlie small-vessel LCV in susceptible individuals [6].

However, in real-world clinical settings, distinguishing between ICI-related vasculitis and other severe drug eruptions is often challenging due to overlapping clinical and histopathologic features. Purpuric eruptions, erythema multiforme/SJS-spectrum reactions, and other severe cutaneous adverse drug reactions may present with similar morphology, particularly when biopsy is delayed or obtained after initiation of systemic corticosteroids.

In this case, the clinical presentation initially raised concern for cutaneous vasculitis due to the presence of palpable purpura. However, histopathologic findings demonstrated full-thickness epidermal necrosis with only mild superficial perivascular lymphocytic infiltrate, without neutrophilic infiltration, leukocytoclasia, or fibrinoid necrosis, making classic LCV unlikely. Instead, these findings are more consistent with a severe drug eruption or an undifferentiated inflammatory cutaneous process.

Importantly, the temporal relationship between empagliflozin initiation and rash onset (within 48 hours of first exposure) represents a strong association and must be considered a significant competing etiology. In contrast, pembrolizumab exposure preceded the rash by a longer interval and occurred in the context of concurrent corticosteroid therapy, further complicating causal attribution.

Several factors limited definitive classification in this case. First, the biopsy was performed approximately three weeks after rash onset. Second, the patient was receiving systemic corticosteroids at the time of biopsy, which may have attenuated diagnostic inflammatory features. Third, DIF was not performed because the specimen was placed in formalin. Finally, the presence of multiple potential offending agents, particularly empagliflozin and pembrolizumab, introduces significant diagnostic uncertainty.

A structured causality assessment (e.g., Naranjo scale) was considered; however, it was not applied due to important limitations in this clinical context, including multiple concurrent drug exposures, lack of drug rechallenge, and confounding effects of ongoing corticosteroid therapy. These factors reduce the reliability of formal causality scoring in complex oncology patients.

Therapeutic management includes discontinuation of the suspected offending agents and initiation of systemic corticosteroids, typically at moderate to high doses, which frequently lead to resolution within weeks. In resistant or systemic cases, steroid-sparing agents such as colchicine, dapsone, and azathioprine, or immunosuppressive therapies like rituximab, tocilizumab, or cyclophosphamide, may be necessary [7]. Our patient's favorable response to corticosteroids, with complete resolution of cutaneous lesions, underscores the importance of early intervention.

From a clinical perspective, this case highlights several important diagnostic pitfalls. Delayed biopsy, prior corticosteroid exposure, and lack of DIF can significantly limit diagnostic accuracy. Additionally, in patients receiving multiple therapies, distinguishing between potential drug-related etiologies can be particularly challenging.

From a clinical standpoint, this case emphasizes the need for prompt dermatologic consultation and early biopsy when new purpura or atypical rashes develop in patients receiving ICIs. When vasculitis is in the differential diagnosis, a fresh lesion should be sampled, and tissue for DIF should be sent in an appropriate transport medium.

Rather than representing a definitive example of pembrolizumab-induced LCV, this case is best interpreted as an undifferentiated purpuric and desquamative drug eruption with competing etiologies occurring in a complex therapeutic setting. Recognizing these diagnostic limitations is essential to avoid misclassification and to guide appropriate management in oncologic patients.

No proprietary scoring systems or licensed clinical assessment tools were used in this case report. The reported stage IIIA lung adenocarcinoma classification was based on the AJCC 8th edition staging system [8].

## Conclusions

This case represents an undifferentiated purpuric drug eruption with competing etiologies, including pembrolizumab and empagliflozin. Nonspecific histopathology, delayed biopsy, lack of DIF, and concurrent corticosteroid use limited diagnostic certainty. The strong temporal association with empagliflozin highlights the importance of considering alternative causes. Recognizing these diagnostic limitations is essential to avoid misclassification in complex oncology patients.
